# NLRP3 inflammasome in rosmarinic acid-afforded attenuation of acute kidney injury in mice

**DOI:** 10.1038/s41598-022-04785-z

**Published:** 2022-01-25

**Authors:** Juheb Akhter, Jasim Khan, Madhu Baghel, Mirza Masroor Ali Beg, Poonam Goswami, Mohd Amir Afjal, Shahzad Ahmad, Haroon Habib, Abul Kalam Najmi, Sheikh Raisuddin

**Affiliations:** 1 Molecular Toxicology Laboratory, Department of Medical Elementology and Toxicology, Jamia Hamdard (Hamdard University), New Delhi, 110062 India; 2 Department of Pharmacology, School of Pharmaceutical Education and Research, Jamia Hamdard (Hamdard University), New Delhi, India; 3 Metabolic Research Laboratory, National Institute of Immunology, New Delhi, 110067 India; 4 Present Address: School of Medicine, University of Alabama at Birmingham, Birmingham, AL 35233 USA

**Keywords:** Medical research, Nephrology

## Abstract

Cisplatin (CP) is a well-known anticancer drug used to effectively treat various kinds of solid tumors. CP causes acute kidney injury (AKI) and unfortunately, there is no therapeutic approach in hand to prevent AKI. Several signaling pathways are responsible for inducing AKI which leads to inflammation in proximal convoluted tubule cells in the kidney. Furthermore, the nucleotide-binding oligomerization domain (NOD)-like receptor containing pyrin domain 3 (NLRP3) inflammasome is involved in the CP-induced AKI. In this study, we investigated therapeutic effects of rosmarinic acid (RA) against inflammation-induced AKI. RA was orally administered at the dose of 100 mg/kg for two consecutive days after 24 h of a single injection of CP at the dose of 20 mg/kg administered intraperitoneally in Swiss albino male mice. Treatment of RA inhibited the activation of NLRP3 signaling pathway by blocking the activated caspase-1 and downstream signal molecules such as IL-1β and IL18. CP activated HMGB1-TLR4/MyD88 axis was also found to be downregulated with the RA treatment. Activation of nuclear factor-κB and elevated protein expression of cyclooxygenase-2 (COX-2) were also found to be downregulated in RA-treated animals. Alteration of early tubular injury biomarker, kidney injury molecule-1 (KIM-1), was found to be subsided in RA-treated mice. RA has been earlier reported for antioxidant and anti-inflammatory properties. Our findings show that blocking a critical step of inflammasome signaling pathway by RA treatment can be a novel and beneficial approach to prevent the CP-induced AKI.

## Introduction

Cisplatin (CP) treatment has been shown to be an effective therapeutic approach against malignancies of ovary, head, bladder, lung, neck, testis and cervix^1^. However, adverse side effects such as nephrotoxicity, neurotoxicity and ototoxicity hinder its clinical uses^1^. Approximately, 25–30% of the patients receiving CP treatment showed acute kidney injury (AKI)^2^. CP and its metabolites get absorbed into the renal tubular cells via organic cation transporters (OCT) found at the basolateral side of the renal tubular cells where they get accumulated and cause renal tubular cell death and AKI^3^. The mechanisms behind CP-induced AKI include tubular necrosis, inflammation, apoptosis and oxidative stress leading to severe nephropathies^4^.

Rosmarinic acid (RA) (IUPAC name—3-(3,4-dihydroxyphenyl)-2-{[(2E)-3-(3,4-dihydroxyphenyl)prop-2-enoyl]oxy}propanoic acid is a widely distributed water soluble polyphenolic natural compound mainly found in plants such as *Perilla frutescens* (family Lamiaceae), *Sarcandra glabra* (family Chloranthaceae) and *Rosmarinus officinalis* (family Lamiaceae). RA has been reported for its broad range of biological activities and as an antioxidant, anti-inflammatory, anti-allergic, antibacterial, antiviral and anti-apoptotic agent^5–10^. Phenolic structure of RA is responsible for its strong antioxidant activity, as it can easily donate its electrons or hydrogen atoms to counterbalance the oxidative radicals^11^. Number and the position of hydroxyl groups denote the antioxidant activity of phenolic compounds. A phenolic compound such as RA having hydroxyl groups on the *ortho* position of the aromatic ring can greatly enhance its antioxidant properties^12^. Because of its strong antioxidant activity, RA is a good drug candidate for the treatment of oxidative stress-induced pathological conditions. Previous studies have shown the ameliorative effect of RA on the acute liver toxicity in mice by mitigating the inflammation and oxidative stress^13,14^.

Kidney injury molecule-1 (KIM-1) is a type I transmembrane glycoprotein belonging to the immunoglobulin family and is barely expressed in the healthy kidney tissue. However, it is overexpressed in the proximal tubule epithelial cell injury indicating acute kidney injury. KIM-1 is a specific early renal tubular injury biomarker with histological changes showing AKI and kidney dysfunction^15,16^.

Inflammasomes are intracellular protein complexes activated upon infections or stresses. To date five inflammasomes have been clearly identified and the best studied and characterized one is NLRP3 inflammasome^17,18^. NLRP3 inflammasome consists of nucleotide-binding oligomerization domain (NOD)-like receptor containing pyrin domain 3 (NLRP3), apoptosis-associated speck-like protein containing a caspase recruitment domain (ASC) and procaspase-1^18^. NLRP3 inflammasome complex cleaves pro-caspase-1 into an active form to mediate the maturation of pro-inflammatory cytokines including interleukin (IL)-1β and IL-18^19^. Toll-like receptor 4 (TLR4) is also activated in response to kidney damage, which activates a variety of downstream signal transduction pathways including the activation of NLRP3 inflammasome^20^. In the initial stages of CP-induced AKI, the proximal renal tubular cell death also causes inflammation that amplifies the kidney injury^21^. Cellular toxic responses such as oxidative stress and necrosis promote the release of high mobility group box 1 (HMGB1) which further causes the inflammatory response through pattern recognition receptors such as TLR4^22^. CP participates in the release of HMGB1 from the renal proximal tubular cells which contribute in the enhancement of the inflammation during the kidney injury^22^. Increased expression of oxidative stress markers and decrease in the activity of antioxidant enzyme (superoxide dismutase; SOD) are observed with the oxidative stress in the renal proximal tubular epithelial cell in the AKI that lead to the activation of NLRP3 inflammasome and proinflammatory pathways including nuclear factor-κB (NF-κB)^23,24^. The toll/IL-1 receptor (TIR) family includes IL-1R1, a cell surface receptor, which further activates the NF-κB and MAPK pathways via signaling through myeloid differentiation primary response protein 88 (MyD88) and triggers the activation of the pro-inflammatory signaling pathway^25^. Under various physiological conditions NF-κB and COX-2 participate in the regulation of cell signaling and play a critical role in CP-induced AKI in mice^26^. It has been reported that the NF-κB pathway depending on the degradation of IκB becomes activated with CP treatment and increases the release of inflammatory cytokine series including TNF-α, IL-1β and TGF1β^27^. It is also reported that the upregulation of IL-6 is the hallmark of activation of NFκB signaling and inflammation^28,29^.

We studied CP-induced AKI and its modulation by RA and associated molecular mechanisms and report data on the following aspects: (i) AKI induced by a single injection of CP was investigated, (ii) NLRP3 inflammasome signaling pathway activation was evaluated in CP-induced AKI and (iii) the therapeutic effect of RA, as a caspase-1 inhibitor against CP-induced AKI was examined.

## Results

### Effect of RA on CP-induced alterations in blood urea nitrogen (BUN) and serum creatinine (Cr)

We found significantly (*p* < 0.001) elevated levels of serum Cr and BUN in CP-treated animals compared to controls (CON) (3.11 ± 0.32 folds for Cr and 4.16 ± 0.31 folds for BUN). Treatment with RA in CP + RA100 group significantly (*p* < 0.05) decreased the Cr and BUN levels (1.47 ± 0.16 folds for Cr and 3.00 ± 0.36 folds for BUN) (Table 1). No significant difference between levels of controls and RA treatment group was observed.

**Table 1:** Effect of RA on CP-induced changes in kidney function markers.

Parameter	CON	Group CP	CP + RA100	RA100
BUN	1.00	4.16 ± 0.31***	3.00 ± 0.36^#^	0.94 ± 0.08
Creatinine	1.00	3.11 ± 0.32***	1.47 ± 0.16^#^	1.03 ± 0.13

All the data were calculated as fold values as compared with each group. CP-treated animals showed significantly (***p < 0.001) higher levels of BUN and serum Cr when compared with control group of animals. However, treatment with RA at the dose of 100 mg/kg significantly (^#^*p* < 0.05) reduces the level of BUN and Cr in CP + RA100 group of animals when compared with CP group of animals. No significant changes in the level of BUN and serum Cr were observed in RA100 treated group of animals vs. controls (*n* = 6).

### Effect of RA on histopathological changes in CP-induced AKI

**Figure 1:**
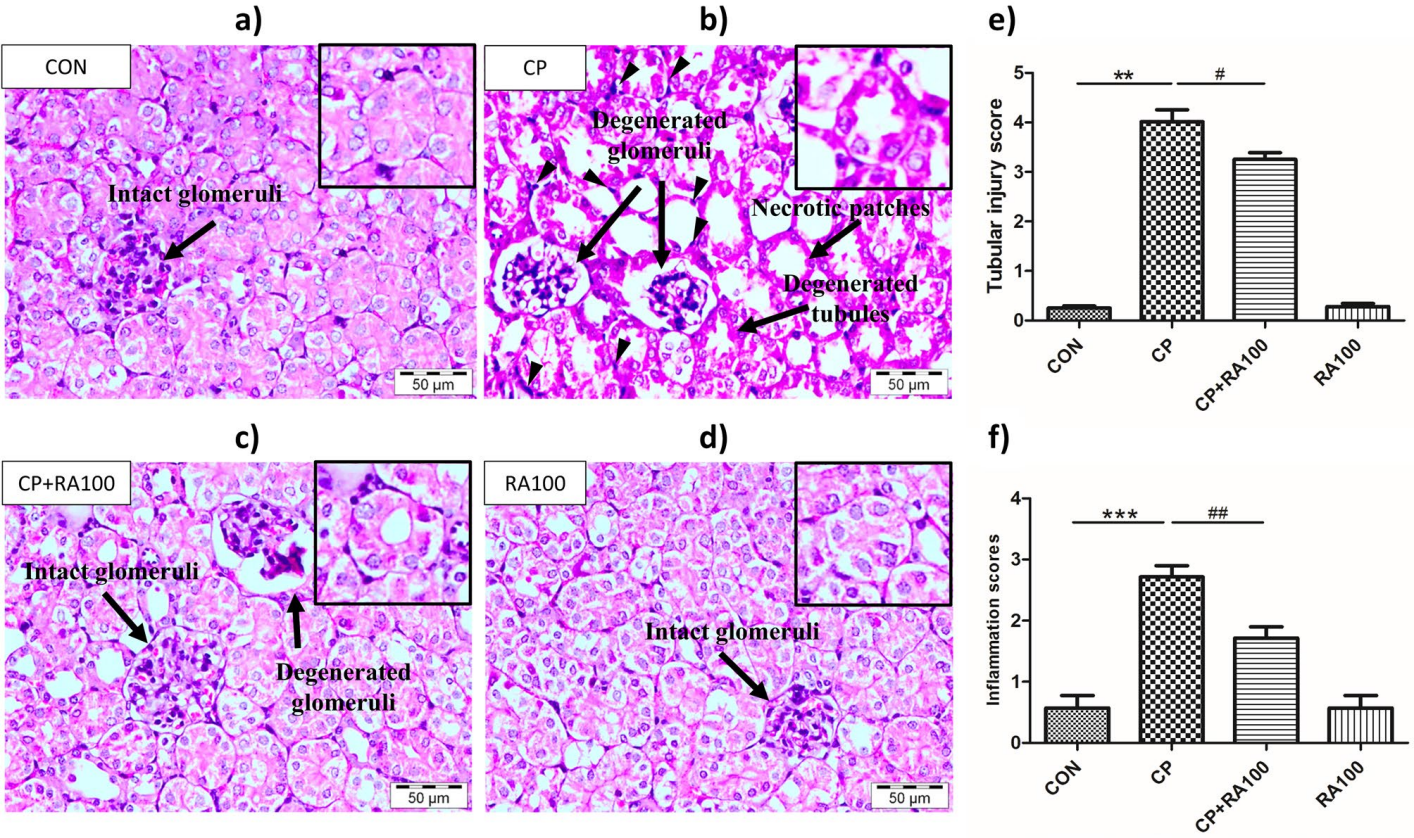
Effect of RA on kidney histological morphology changes in various groups of animals. In (**a**), kidney section of control group animal treated with normal saline shows normal histoarchitecture with smooth and homogenous cellular structure. Normal glomeruli are represented by a black arrow. In (**b**), kidney section of CP-treated mouse shows altered histology with necrotic changes, degenerated glomeruli and tubular damage represented by the black arrows. In (**c**), kidney section of mouse treated with RA along with CP depicts a significant restoration in histology. However, some degenerated glomeruli still persisted with intact and normal glomeruli. (**d**) RA alone treatment showed no adverse changes in histology of mouse kidney section. In (**e**), histogram represents significant changes after CP and RA administration in mouse kidney sections and (**f**) histogram represents the inflammation scores. Treatment of CP caused a significant increase in histological changes (**p < 0.01) and significant infiltration of inflammatory cells (***p < 0.001) when compared with the control group of animals. Treatment of RA attenuated the CP-induced tubular injury and inflammation in a significant manner (^**#**^*p* < 0.05) and (^*##*^*p* < 0.01), respectively in CP + RA group when compared with the CP-treated group. Scale bars: 50 μm; original magnification: × 40.

As shown in Fig. 1a,d, normal tubular morphology was observed in renal cortex and medulla in control and RA group mice. Mice treated with CP at the dose of 20 mg/kg showed pronounced histopathological changes in the cortico-medullary junction including degenerated tubules with accumulation of cellular debris in the lumen, necrotic patches in the cortex region along with diffused hemorrhagic areas, degenerated glomeruli with enlarged Bowmen’s space and mild hemorrhage diffused throughout the parenchyma. CP group (Fig. 1b) also showed tubular injury and widespread infiltration of inflammatory cells around the renal tubules. In Fig. 1c, treatment with RA in CP + RA100 treatment group at dose of 100 mg/kg showed significant (*p* < 0.05, analysis presented in Fig. 1e) improvement of kidney histoarchitecture with reduction in degenerated glomeruli, necrotic patches and degenerated tubules. Figure 1e represents the statistical analysis of histopathological changes in mouse kidney tissue after CP and RA administration. Figure 1f represents the inflammation scores of each group. CP-treated animals showed a significant (*p* < 0.001) increase in the infiltration of inflammatory cells when compared with CON group animals whereas treatment with RA showed significantly (*p* < 0.01) low infiltration of inflammatory cells in CP + RA100 group.

### Immunohistochemical findings on NLRP3, caspase-1 and IL-1β

**Figure 2:**
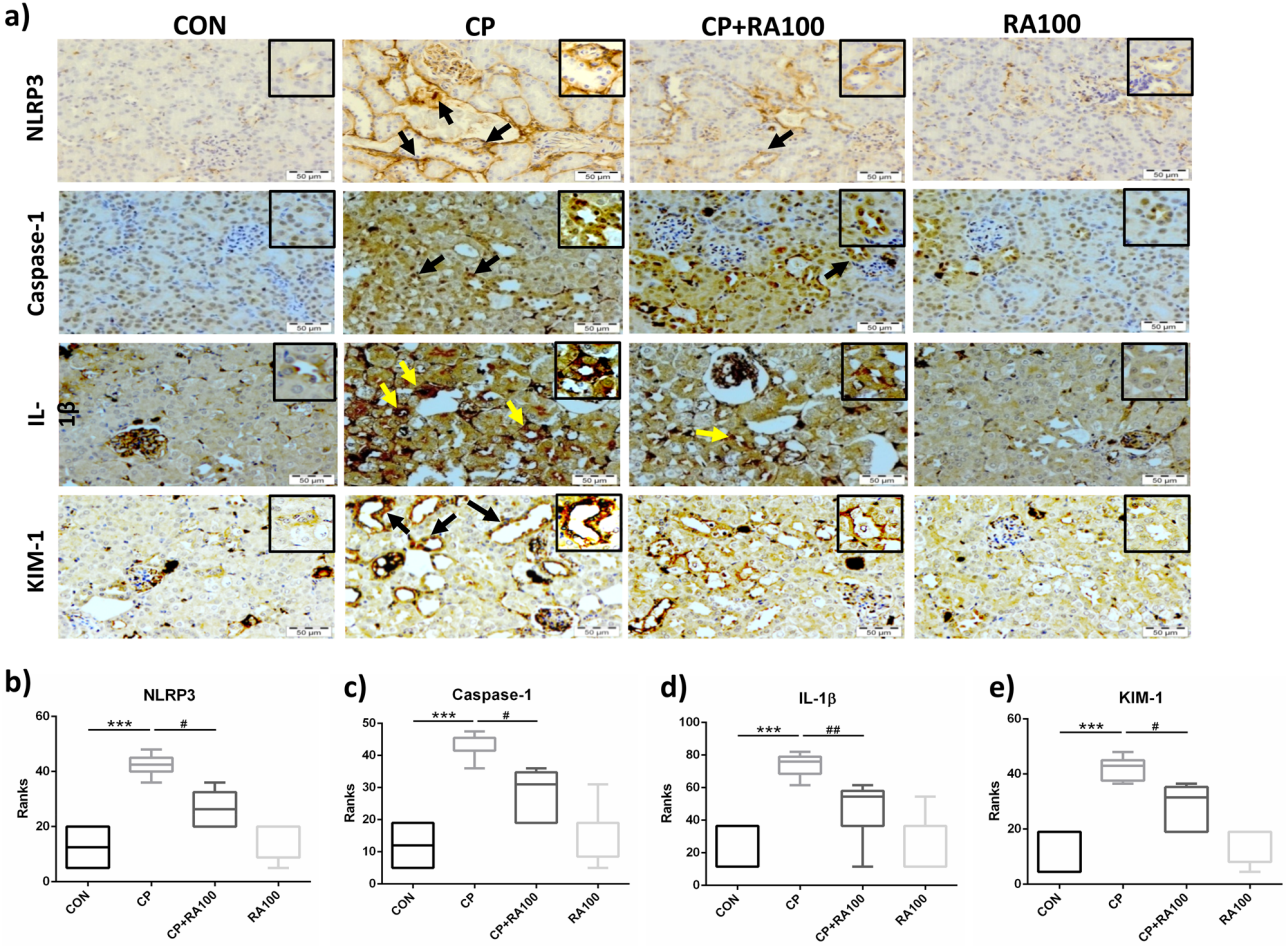
Immunohistochemical analysis of NLRP3, caspase-1, IL-1β and KIM-1 expression after the administration of CP and RA. In (**a**), dark brown staining represents an increase in the expressions of NLRP3, caspase-1, IL-1β and KIM-1 protein as a result of adverse effects of CP in kidney tissue when compared with control group of animals. On the other hand, faint brown color indicates positive anti-inflammatory effect of RA against CP toxicity in CP + RA100 treated group when compared with CP alone group. There was no remarkable change in RA alone treated group of animals when compared with control group of animals. Histograms in (**b**–**e**) represent significant differences in the expression of NLRP3, caspase-1, IL-1β and KIM-1 proteins, respectively after CP and RA treatments. The administration of CP caused a significant increase in the expressions of NLRP3 (***p < 0.001) (**b**), caspase-1 (***p < 0.001) (**c**), IL-1β (***p < 0.001) (**d**) and KIM-1 (***p < 0.001) (**e**) when compared with control group of animals. However, treatment of RA along with CP showed its protection via attenuating these changes significantly in NLRP3 (^**#**^*p* < 0.05), caspase-1 (^**#**^*p* < 0.05), IL-1β (^**##**^*p* < 0.01) and KIM-1 (^**#**^*p* < 0.05) when compared with the CP-treated animals. No significant change was observed in RA only treated animals. Interquartile ranges are shown by a box and the median value is indicated by a line across the box. Statistical analysis was performed using Kruskal–Wallis tests (ANOVA on Ranks). Scale bars: 50 μm, original magnification: × 40.

Pictorial immunohistochemical expression of NLRP3, caspase-1 and IL-1β in different groups is presented in Fig. 2a. It shows an intense immunostaining of respective proteins of interest in treated groups as against controls. Negligible NLRP3, caspase-1 and IL-1β immunopositive staining was observed in RA100 group compared with CON group. Graphs in Fig. 2b–d show immunoreactive scoring (IRS) analyzed by the Kruskal–Wallis test (ANOVA on Ranks) followed by Dunn’s multiple comparison test. There was a significantly (*p* < 0.001) higher expression of NLRP3, caspase-1 and IL-1β proteins in CP treated group when compared with the control group of animals while treatment with RA in CP + RA100 group showed significantly (*p* < 0.05), (*p* < 0.05 and *p* < 0.01) reduced expression of NLRP3, caspase-1 and IL-1β, respectively when compared to only CP treated animals. No changes were noted in immunopositive staining pattern in RA100 treated animals.

### Immunohistochemical finding on AKI biomarker

Pictorial immunohistochemical expression of AKI biomarker KIM-1 is represented in Fig. 2a. Figure 2e graph shows a significant (*p* < 0.001) increase in the expression of KIM-1 protein in CP-treated animals compared with CON group. However, a negligible KIM-1 immunostaining was found in RA100 group compared with CON group. Treatment with RA in CP + RA100 group showed significantly (*p* < 0.05) reduced expression of KIM-1 when compared with only CP-treated animals. No difference in immunostaining was noted in RA100 treated animals compared with control group animals.

### m-RNA expression of NLRP3 inflammasome linked proinflammatory genes

**Figure 3:**
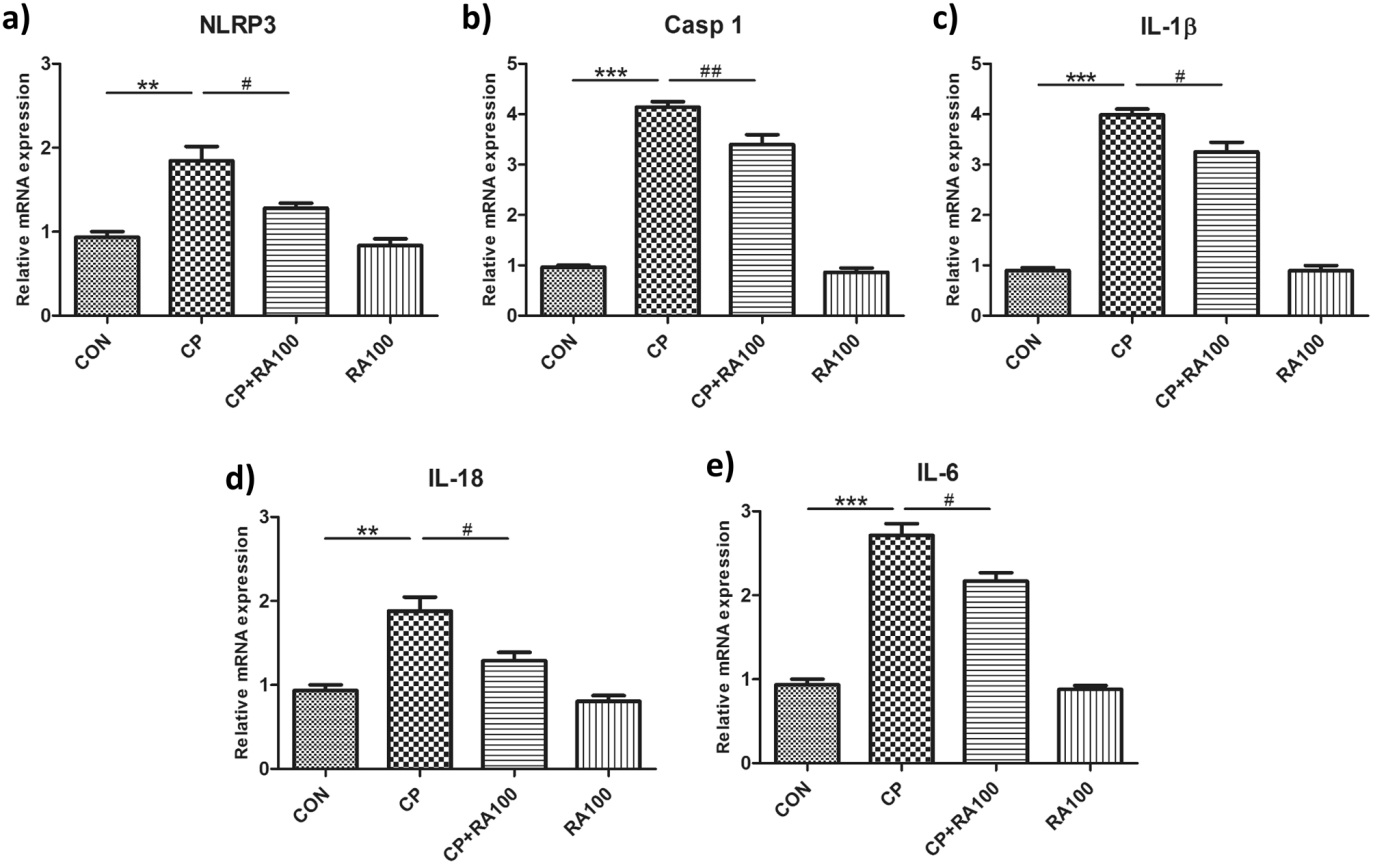
mRNA expression analysis of *NLRP3, caspase-1, IL-1β, IL-18* and *IL-6* in the kidney tissue after the administration of CP and RA. The treatment of CP at the dose of 20 mg/kg caused a significant upregulation in the expression of *NLRP3* (**p < 0.01) (**a**), *caspase-1* (***p < 0.001) (**b**), *IL-1β* (***p < 0.001) (**c**), *IL-18* (**p < 0.01) (**d**) and *IL-6* (***p < 0.001) (**e**) at mRNA levels when compared with control group of animals. On the other hand, the administration of RA at the dose of 100 mg/kg caused a significant down-regulation in the expression of *NLRP3* (^#^*p* < 0.05), *caspase-1* (^##^*p* < 0.01), *IL-1β* (^#^*p* < 0.05), *IL-18* (^#^*p* < 0.05) and *IL-*6 (^#^*p* < 0.05) at mRNA level when compared with CP-treated group of animals. There was no significant difference in RA only treated animals when compared with control group of animals. Each value is represented as mean ± SEM (*n* = 6).

Effect of RA on the mRNA levels of proinflammatory *NLRP3*, *caspase-1*, *IL-1β*, *IL-18* and *IL-6* genes is shown in Fig. 3a–e. The m-RNA expression of *NLRP3* (Fig. 3a), *caspase-1* (Fig. 3b) and *IL-1β* (Fig. 3c) was found upregulated with 1.8, 4.1 and 3.9 folds, respectively in CP-treated mice when compared with control mice. However, m-RNA expression of *NLRP3*, *caspase-1* and *IL-1β* was found to be reduced with 1.2, 3.3 and 3 folds, respectively in CP + RA100 treated mice when data were compared with CP-treated mice. Also, the m-RNA expression of *IL-18* (Fig. 3d) and *IL-6* (Fig. 3e) genes was found upregulated with 1.8 and 2.7 folds in CP-treated mice when compared with control mice. However, the m-RNA expression of *IL-18* and *IL-6* genes was found to be reduced with 1.2 and 2.1 folds, respectively in CP + RA100 treated mice in comparison to CP-treated animals. The m-RNA expression results revealed that expression of proinflammatory genes in CP-treated mice was down-regulated in CP + RA100-treated mice in a significant manner (*p* < 0.05 in case of *NLRP3*, *IL-1β*, *IL-18* and *IL-6* and *p* < 0.01 in case of *caspase-1*).

### Effect of RA treatment on the assembly of NLRP3 inflammasome proteins against the CP-induced AKI in western blotting

**Figure 4:**
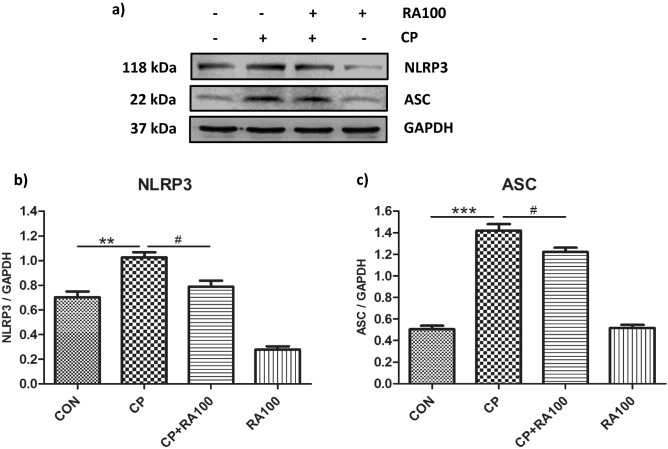
Western blot analysis and densitometric quantification of hallmarks of NLRP3 inflammasome activation, NLRP3 and ASC proteins in the mouse kidney treated with CP and RA. (**a**) Represents the immunoblots of NLRP3 and ASC proteins in CP and RA treatments in mouse kidney. The administration of CP at the dose of 20 mg/kg b.wt. caused an increase in the expression of NLRP3 and ASC proteins when compared with control group of animals. While the treatment with the dose of 100 mg/kg b.wt. of RA along with CP caused a decrease in the expression of NLRP3 and ASC proteins when compared with CP alone treated group of mice. In this figure histogram (**b**) and (**c**) represent significant differences between CP and CP + RA100 treated group of animals. The treatment of CP caused a significant increase in the expression of NLRP3 (**p < 0.01) and ASC (***p < 0.001) proteins when compared with control group of animals. However, the treatment of RA along with CP caused a significant restoration in the expression of NLRP3 (^#^*p* < 0.05) and ASC (^#^*p* < 0.05) proteins when compared with CP alone treated group of mice. No significant differences were noticed in RA alone treated group of animals when compared with the control group of animals. Data are expressed as means ± SEM (*n* = 6).

**Figure 5:**
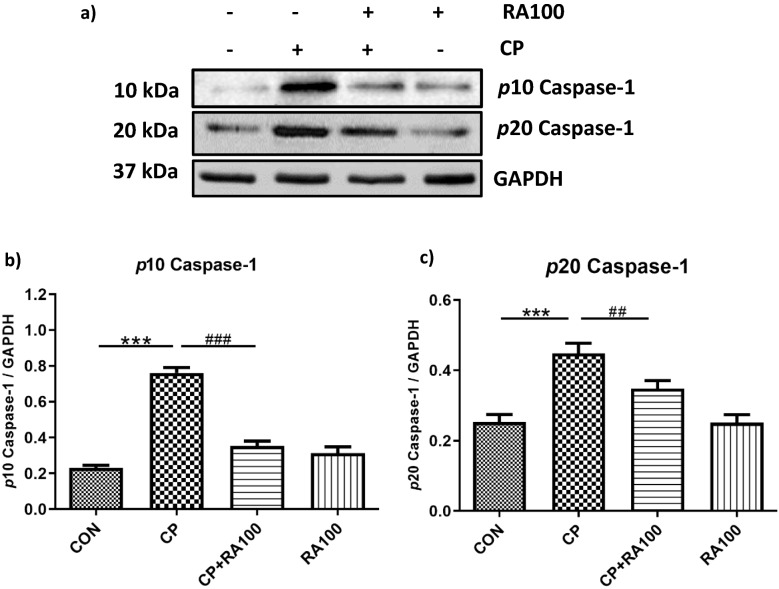
Analysis of activated caspase-1 by western blotting after CP and RA administration in mouse kidney. (**a**) Represents the immunoblots of activated cleaved subunits (*p*10 caspase-1 and *p*20 caspase-1) of caspase-1 after CP and RA treatments. The administration of CP caused an increase (dark bands) in the expression of cleaved *p*10 caspase-1 and *p*20 caspase-1 proteins when compared with control group of animals. While the treatment of RA along with the administration of CP caused a decrease (light bands) in the expression of *p*10 caspase-1 and *p*20 caspase-1 proteins when compared with the CP alone treated group of mice. Histogram (**b**) and (**c**) represent significant differences between CP and CP + RA100 treated group of animals. The treatment of CP caused a significant increase in the expression of *p*10 caspase-1 (***p < 0.001) and *p*20 caspase-1 (***p < 0.001) proteins when compared with control group of animals. However, the treatment of RA along with CP caused a significant restoration in the expression of *p*10 caspase-1 (^###^*p* < 0.001) and *p*20 caspase-1 (^##^*p* < 0.01) proteins when compared with CP alone treated group of mice. No significant differences were noticed in RA alone treated group of animals when compared with control group of animals. Data are expressed as means ± SEM (*n* = 6).

We studied preventive effect of RA treatment on activated NLRP3 signaling pathway proteins such as NLRP3 (Fig. 4a,b), ASC (Fig. 4a,c), and both activated subunits of caspase-1 (Fig. 5a–c). Statistical analysis revealed a significant increase in the protein expression of NLRP3 (*p* < 0.01), ASC (*p* < 0.001) and both activated cleaved subunit of caspase-1 (*p* < 0.001) in CP-treated (20 mg/kg) mice as compared with control mice. CP + RA treatment group mice showed a significantly decreased expression of NLRP3 (*p* < 0.05), ASC (*p* < 0.05), *p*10 caspase-1 (*p* < 0.001) and *p*20 caspase-1 (*p* < 0.01) as compared to CP-treated mice.

### RA administration reduced the expression of proinflammatory COX-2 and NFκB-p65 proteins

**Figure 6:**
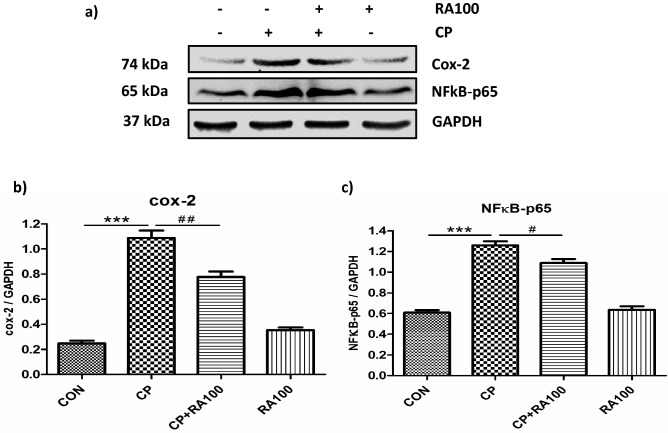
Western blotting analysis of inflammatory markers. Effect of RA and CP on the expression of COX-2 and NFκB-p65 in mouse kidney tissue as studied by western blot analysis is shown in (**a**) by immunoblots of inflammatory markers COX-2 and NFκB-p65. The administration of CP caused an increase in the expression of COX-2 and NFκB-p65 proteins when compared with control group of animals. While the treatment of RA along with the administration of CP decreases the expression of COX-2 and NFκB-p65 proteins when compared with CP alone treated group of mice. In this figure histograms (**b**) and (**c**) represent significant differences between CP and CP + RA100 treated group of animals. The treatment of CP caused a significant increase in the expression of COX2 (***p < 0.001) and NFκB-p65 (***p < 0.001) proteins when compared with control group of animals. However, the treatment of RA along with CP caused a significant restoration in the expression of COX-2 (^##^*p* < 0.01) and NFκB-p65 (^#^*p* < 0.05) proteins when compared with CP alone treated group of mice. No significant differences were noticed in RA alone treated group of animals when compared with control group of animals. Data are expressed as means ± SEM (*n* = 6).

Expression pattern of COX-2 protein is shown in Fig. 6a,b and that of NFκB-p65 protein in Fig. 6a,c. CP-treated mice showed a significant increase (*p* < 0.001) in the expression of both COX-2 and NFκB-p65 proteins as compared to control mice. A significant decrease in the expression of COX-2 (*p* < 0.01) and NFκB-p65 (*p* < 0.05) proteins was observed in RA-treated mice as compared to CP-treated mice. No significant change was noted in only RA-treated mice when data were compared with control group of mice.

### RA downregulates the HMGB1-TLR4/MyD88 axis in CP-induced AKI

**Figure 7:**
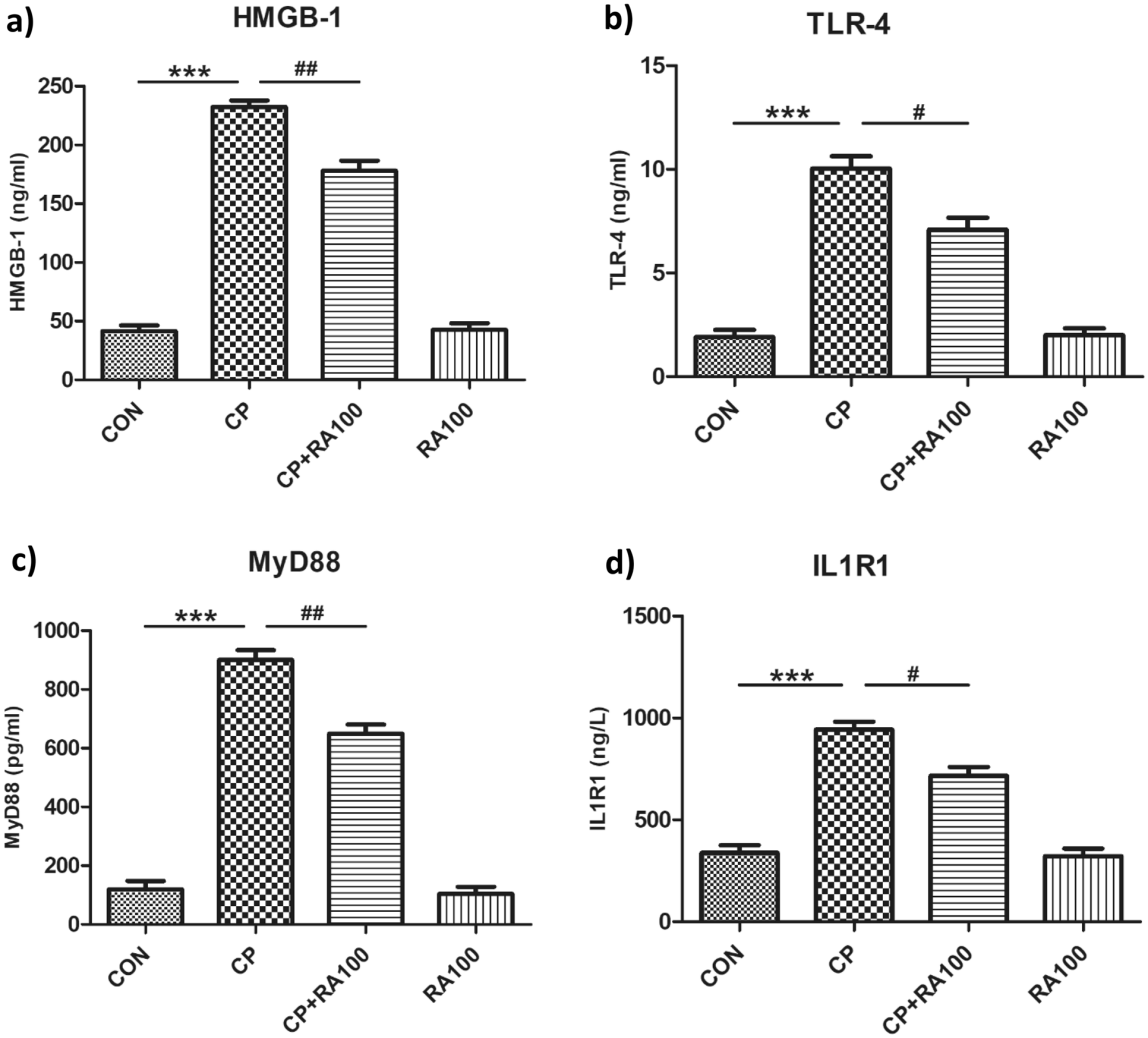
Effect of RA and CP treatments on the expression levels of HMGB-1, TLR-4, MyD88 and IL-1R1 proteins. In figure histograms (**a**)–(**d**) represent significant changes in the expression level of HMBG-1, TLR-4, MyD88 and IL-1R1 proteins, respectively measured by ELISA. The administration of CP caused a significant increase in the level of HMBG-1 (***p < 0.001) (**a**), TLR-4 (***p < 0.001) (**b**), MyD88 (***p < 0.001) (**c**) and IL-1R1 (***p < 0.001) (**d**) at the dose of 20 mg/kg b.wt. in mouse kidney when compared with control group of animals. However, administration of RA at the dose of 100 mg/kg b.wt. caused a significant restoration of the expression level of HMBG-1 (^##^*p* < 0.01), TLR-4 (^#^*p* < 0.05), MyD88 (^##^*p* < 0.01) and IL-1R1 (^#^*p* < 0.05) protein levels when compared with CP alone group of animals. There was no significant change in RA alone treated animals when compared with control group of animals. Each value is represented as mean ± SEM (*n* = 6).

Expression levels of HMGB-1, TLR-4, MyD88 and IL-1R1 proteins in mouse kidney homogenate were determined by using ELISA kits (Fig. 7). CP administration at the dose of 20 mg/kg b.wt. caused a significant increase in the expression of HMGB-1 (*p* < 0.001) (Fig. 7a), TLR-4 (*p* < 0.001) (Fig. 7b), MyD88 (*p* < 0.001) (Fig. 7c) and IL-1R1 (*p* < 0.001) (Fig. 7d) in mouse kidney when compared with the control group of animals. However, treatment of RA at the dose of 100 mg/kg caused a significant reduction in the expression of HMGB-1 (*p* < 0.01), TLR-4 (*p* < 0.05), MyD88 (*p* < 0.01) and IL-1R1 (*p* < 0.05) in mouse kidney when compared with the CP-treated animals. No significant changes were found in the HMGB-1, TLR-4, MyD88 and IL-1R1 expression in the mouse kidney of group treated only with RA at 100 mg/kg when compared with the control group of animals.

## Discussion

CP-based chemotherapy is used in the patients suffering from many types of solid tumors^30^. CP is an effective and a frequently used anticancer drug, despite its various side effects including nephrotoxicity^4^. The toxicity of CP is caused mainly through inflammation, oxidative stress and apoptosis^31^. Therefore, combinational chemotherapy aiming at multiple mechanisms including reduction in the production of free radicals, and anti-inflammatory and cytoprotective activities prevent CP-induced nephrotoxicity^32^.

Natural compounds from plants such as *Satureja hortensis* (family Lamiaceae) widely used as dietary supplements show beneficial effects against toxicity of xenobiotics and disease-related pathologies directly linked to vital organs^33^. Recent studies have demonstrated that RA possesses an impactful anti-inflammatory and anti-apoptotic ability by blocking the caspase-1 activity^34^. It is reported that RA ameliorated CP-induced ototoxicity without affecting its anticancer activity. It was also demonstrated that RA targeted caspase-1^34^. The inflammasome, which is a protein assembly similar to apoptosome gets activated when caspase-1 becomes part of this assembly in the inflammatory response^35^. Also, caspase-1 plays a key role by converting inactive pro-IL-1β and pro-IL-18 to active proinflammatory cytokines^34^. It has been shown that RA inhibits the induction of iNOS and COX-2 expression and production of pro-inflammatory cytokines such as IL-6, IL-22 and IL-1β in DSS (dextran sulphate sodium)-induced colitis in the mouse model^36^. The present study also showed for the first time that RA inhibited activation of NLRP3 inflammasome signaling pathway by targeting caspase-1 in CP-induced AKI. Guo et al*.*^22^ suggested that pretreatment of HQH (Huaiqihuang) extractum protects the kidney against cisplatin-induced inflammation through the down regulation of HMGB1/TLR4/NFκB pathway. Lv et al*.*^37^ have also demonstrated that RA down-regulates the elevated levels of HMGB1, TLR4 and MyD88 and provides neuroprotection against the neuroinflammation^37^. In another study, Privratsky et al*.*^38^ reported that IL-1R1 knockout mice show protection of the kidney in CP-induced nephrotoxicity. Our study reveals for the first time that the treatment of RA significantly decreases the level of upregulated HMGB1, TLR-4, MyD88 and IL-1R1 proteins in CP-induced AKI offering a molecular mechanism of RA-induced protection against CP toxicity.

Role of inflammatory mediators has earlier been reported in the pathogenesis of CP-induced AKI^26,39,40^. In our study, we observed a significant activation of NLRP3 inflammasome signaling pathway in the CP-induced AKI. We observed that RA protected animals against CP-induced AKI through blockage of caspase-1 of the NLRP3 inflammasome signaling pathway. CP-induced AKI involves a series of complex multi-factorial processes, and inflammation is considered as an important factor^41^. In response to kidney injury, inflammation is triggered by the resident macrophages and dendritic cells that leads to amplification in the infiltration of leukocytes by which monocytes, lymphocytes and neutrophils contribute to the evolution of kidney injury^42^. CP-induced inflammatory cascade elevates levels of cytokines such as IL-6 and IL-18 inside the kidney and the elevated level of these cytokines can be used as a diagnostic marker for the detection of the severity of the kidney damage^43,44^. Therefore, down-regulation of the inflammatory response may be an effective approach against the CP-induced AKI and associated pathologies. In the current study, we noted an upregulation of NLRP3 inflammasome and pro-inflammatory cytokines after single injection of CP. Severely damaged renal tubular epithelium accompanied these changes. Importantly, in RA-treated animals the expression of both activated cleaved subunits of caspase-1 (*p*10 caspase-1 and *p*20 caspase-1) and IL-1β proteins was found to be significantly decreased as compared to CP-treated mice. Also, CP-induced AKI involves structural damage in the kidney, specifically tubular injury. KIM-1 is an early biomarker of CP-induced AKI. Its expression was not detected in the normal kidney tissue. However, with the injury of proximal tubule epithelial cells KIM-1 expression was found to be upregulated in CP-induced AKI^15,45^. In this study we also characterize the expression of KIM-1 in renal tissue by using immunohistochemical techniques and found that administration of RA reduced the elevated expression of KIM-1 in CP-induced AKI.

These results suggest that injured tubular epithelial cells may be an important determinant of CP-induced AKI. CP treatment caused ROS-induced effects in animals that included elevated lipid peroxidation (LPO), decreased glutathione reduced (GSH) and decrease in the activity of enzymes with antioxidant role such as superoxide dismutase (SOD) (Supplementary Fig. S1). It may lead to activation of NLRP3 inflammasome signaling. Our results are supported by previously published reports suggesting a prominent role for ROS in triggering the NLRP3 inflammasome^46^. RA has shown an inhibitory effect on the NLRP3 inflammasome resulting in reduced ROS levels^47^. Caspase-1 is one of the key players of the inflammasome signaling and its modulation can be a potential approach to tackle the chemically-induced inflammation^31^. Researchers have shown blockage of caspase-1 by RA suggesting its therapeutic potential against the systemic inflammation^34^. Previously, treatment of MCC950, a small molecule and inflammasome inhibitor showed reduction in the maturation and protein expression of caspase-1, NLRP3 and IL-1β in mice with experimentally-induced non-alcoholic steatohepatitis^48^. Similarly, in the spontaneous lupus nephritis a mutant strain of NZM2328 mice with MCC950 administration showed significant inhibition in the expression of pro-caspase-1, *p*20 caspase-1, NLRP3 and IL-1β^49^. In agreement with these results, RA also significantly inhibited these key proteins of inflammasome signaling. Furthermore, it has been demonstrated earlier that in human leukemia U937 cells, RA caused cell death by down-regulating TNF-α-regulated NF-κB activation and ROS generation^50^. Our results also demonstrated a similar outcome after RA administration, as shown earlier by Domitrović et al.^51^ suggesting RA-induced inhibitory expression pattern of NF-κB and TNF-α. Both NF-κB and TNF-α are considered as hallmarks of inflammation^52,53^. Collectively, our study successfully demonstrated the therapeutic action of RA administration via modulation of inflammasome mediated damage in CP-induced acute kidney injury. Our results strengthen therapeutic application of RA in preventing the CP-induced kidney injury in cancer patients.

## Conclusions

**Figure 8:**
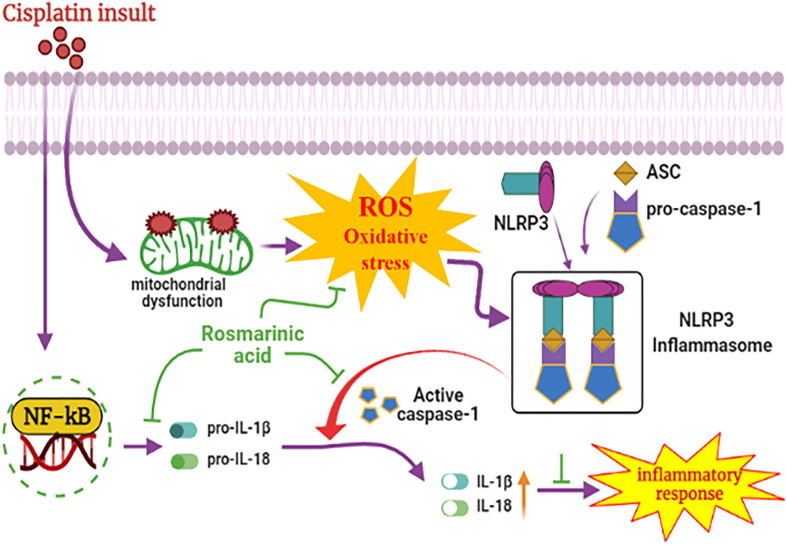
Summarized schematic pathway representation of NLRP3 inflammasome modulation by RA administration in CP-induced mouse AKI model. RA showed anti-inflammatory effects by inhibiting the proinflammatory markers in the mouse kidney.

In conclusion, RA successfully ameliorated CP-induced nephrotoxicity in mice via suppression of key inflammatory markers, and deactivation of inflammasome assembly in the kidney tissue (Fig. 8). These data along with previous results expound role of RA as a promising nephroprotective candidate suitable for the adjuvant therapeutic intervention in the chemotherapy.

## Materials and methods

### Chemicals

Bovine serum albumin (BSA), glycine, protease inhibitor cocktail, RA and Tween 20 were from Sigma-Aldrich (St. Louis, MO, USA). CP was procured from Dr Reddy’s Laboratories (Hyderabad, India). Primary and secondary antibodies were purchased from Cell Signaling Technology, Inc. (Danvers, MA, USA), Santa Cruz Biotechnology, Inc. (Dallas, TX, USA) and Elabscience (Houston, TX, USA). Eosin, hematoxylin, Ponceau S, β-mercaptoethanol and Triton X-100 were purchased from Hi-Media Laboratories Pvt. Ltd. (Mumbai, India). Acrylamide, bisacrylamide, ethylene-diaminetetraacetic acid and tris-hydroxymethylaminomethane were purchased from Sisco Research Laboratories Pvt. Ltd. (Mumbai, India). Horseradish peroxidase was from E. Merck Ltd. (Mumbai, India). Other routine chemicals and reagents were purchased from either Sigma-Aldrich or accredited local vendors.

### Experimental animals

Study was conducted in male Swiss albino male mice (8–10 weeks of age, body weight (b.wt.)—25–30 g) obtained from the Central Animal House Facility of Jamia Hamdard, New Delhi. Animals were housed in polypropylene cages in groups of six per cage under a 12-h/12-h light/dark cycle at 23 °C, 60 ± 10% humidity with food and water *ad libitum*. Before the treatment, mice were acclimatized for one week. Study protocols were duly approved by the Institutional Animal Ethical Committee (Project # 1289) accredited by the Committee for Purpose of Control and Supervision of Experiments on Animals (CPCSEA), Government of India which has adopted ARRIVE Guidelines. All experiments were performed in accordance with relevant guidelines and regulations.

### Treatment regimen

A single injection of CP at the dose of 20 mg/kg in normal saline was administered intraperitoneally (ip) to mice to generate the CP-induced AKI model^45,54–56^. RA at the dose of 100 mg/kg dissolved in water was given to mice orally for two consecutive days after 24 h of CP injection. CP and RA dose schedules were based on previously published reports^45,57,58^. Twenty-four animals were randomly divided into four groups (*n* = 6) as follows.

I. CON: Served as control and administered normal saline (10 ml/kg)—ip.
II. CP: Served as toxicant group and received a single dose of CP (20 mg/kg in normal saline)—ip.
III. CP + RA100: RA at the dose of 100 mg/kg dissolved in water was administered through gavage once a day for two consecutive days after 24 h of ip CP injection.
IV. RA100: Received only RA (100 mg/kg) by gavage.

All animals were anesthetized with pentobarbital (30 mg/kg—ip), subsequently sacrificed at 72 h after CP injection. Kidney was dissected out from each animal for examination of various biochemical parameters and a portion of it was snap-freezed for expression studies. Blood was drawn from the retro-orbital sinus and serum was utilized for serological markers of nephrotoxicity. Initiation of CP-induced AKI was proven by measuring KIM-1 in the serum by using ELISA method (Bioassay Technology Laboratory, Shanghai, China; Cat No. E0617Mo).

### Assessment of renal function biochemical markers

Serum prepared from the blood was used to quantify blood urea nitrogen (BUN) by the method of Kanter^59^ and creatinine (Cr) by the method of Hare^60^.

### Histopathological analysis

Kidney tissue was excised out, fixed in neutral formalin (10%) and embedded in paraffin. Blocks were made and tissues were cut to get sections of 5 µm thickness. Xylene and ethanol were used to make paraffin embedded tissues and then deparaffinized. Phosphate buffer saline (PBS) was used to wash the slides and permeabilized with permeabilization solution (0.1 M citrate and 0.1% Triton X-100). Hematoxylin and eosin (HE) were used to stain the deparaffinized sections. Slides were observed at 40 × magnification under light microscope (OLYMPUS BX51, Olympus, Tokyo, Japan; software—cell^**˄**^F 5.1 version 5.1.2108; http://www.olympuscanada.com/cpg_section/cpg_support) to examine the histo-architecture of renal tissues. Tubular damage was analyzed by scoring necrotic patches, tubular dilation, degenerated tubules, and degenerated glomeruli with enlarged Bowmen’s space in 10 different fields in the cortico-medullary junction of the kidney^61^. Histopathological changes were blindly scored by a pathologist on a 5-point scale: 0 = no damage, 1 = 10%, 2 = 10–25%, 3 = 25–50%, 4 = 50–75%, and 5 = more than 75% of the cortico-medullary junction injured. The histological examination of infiltration based inflammation was scored on a scale from 0 to 3, where 0 = normal/absence of pathology (< 5% pathology), 1 = mild (< 10% pathology), 2 = moderate (15–20% pathology), and 3 = severe (> 20% pathology)^62^.

### Immunohistochemical staining for the detection of inflammatory protein expression

The protective effect of RA on CP-induced inflammation in kidney tissue was also assessed by immunohistochemical staining as per the previously published protocol of Shahid et al.^63^. The kidney tissue was fixed in formalin and embedded in paraffin. Kidney sections of 5 μm thickness were cut onto poly-lysine coated glass slides. The tissue sections were de-paraffinized three times (5 min each) with xylene followed by dehydration in graded ethanol and finally rehydrated in running tap water. Sections were boiled for 5–7 min in 10 mM citrate buffer (pH 6.0) for the antigen retrieval. To minimize the non-specific staining sections were incubated with hydrogen peroxide for 15 min and then rinsed three times (5 min each) with 1 × PBST (0.05% Tween-20). Blocking solution was applied on the sections for 10 min and sections were incubated with diluted rabbit polyclonal primary antibodies, anti-NLRP3 (dilution 1:400), anti-IL-1β (dilution 1:100) and anti-caspase-1 (dilution 1:100) overnight at 4 °C in a humid chamber. Further processing was followed according to the instructions of ULTRA VISION PLUS DETECTION SYSTEM ANTI-POLYVALENT, HRP/DAB (Ready-To-Use) staining kit (Thermo Scientific, Waltham, MA, USA). The peroxidase complex was visualized with 3,3′-diaminobenzidine (DAB) and the slides were counterstained with hematoxylin, dried and covered with DPX mountant for observation under microscope (OLYMPUS BX51) at 40 × magnification.

### Quantitative evaluation of NLRP3, caspase-1, IL-1β and KIM-1

For the quantitative evaluation of NLRP3, caspase-1, IL-1β and KIM-1 the IRS method of Fedchenko and Reifenrath^64^ was used which is based on the scoring of intensity and quantity of immune staining in renal tissue. Following are the staining intensity scoring criteria: 0—no reaction, 1—mild, 2—moderate, and 3—intense and the positive cells percentage was evaluated as 0—no positive cells, 1—< 10% positive cells, 2—10–50% positive cells, 3—51–80% positive cells, and 4—> 80% positive cells. The IRS was calculated by multiplying the staining intensity score and percentage of positive cell score and every tissue sample was classified into negative (IRS points 0–1), mild (IRS point 2, 3), moderate (IRS point 4–8) or strong (IRS point 9–12). All slides were examined by two independent observers who were not aware of the experimental protocol.

### Western blot analysis

Western blotting was performed to check expression of inflammatory protein markers such as COX-2, NFκB-p65 and IL-1β and the markers of inflammasome assembly such as NLRP3, ASC, *p*10 caspase-1 and *p*20 caspase-1 as per the previously published protocol described by Khan et al*.*^65^. The snap-freezed renal tissue was homogenized in RIPA lysis buffer (Sigma-Aldrich) with 1 × protease Inhibitor Cocktail (Sigma-Aldrich) in a Potter–Elvehjem homogenizer on ice and then centrifuged at 10,000×*g* for 15 min at 4 °C. Supernatant was taken for the protein analysis and stored at − 80 °C. Bradford assay^66^ was used for the protein quantification using Gen 5.0 software version 3.0 (https://www.biotek.com/products/software-robotics-software/gen5-microplate-reader-and-imager-software/) provided with 96 well plate reader (BIOTEK POWER WAVE XS2, Winooski, VT, USA). Laemmli loading buffer (6 ×) was added to the protein sample and the equal amount of protein sample was loaded to 10% sodium dodecyl sulphate polyacrylamide gel electrophoresis (SDS-PAGE) gel to be electrophoresed with SDS running buffer and reference bands of broad range pre-stained protein ladder (Genetix Asia Pvt. Ltd, New Delhi, India). To run gel electrophoresis mini-PROTEAN Tetra Cell SDS-PAGE gel running unit (Bio-Rad, Hercules, CA, USA) was used. After the electrophoresis protein was transferred from gel to polyvinylidene difluoride (PVDF) membrane (mdi Advance Micro devices Pvt. Ltd., Ambala, India) in the transfer buffer. Membranes were blocked in 5% (*w*/*v*) skimmed milk made in PBST for 1 h and incubated with primary and then HRP-conjugated IgG secondary antibodies dissolved in 2% BSA in PBST. Details of primary and secondary antibodies used in this study are provided in supplementary table (Table S1). Proteins were detected with enhanced chemiluminescence (ECL) detection kit (Thermo Fisher Scientific) and visualized on CHEMIDOC IMAGING SYSTEM (Syngene, Cambridge, UK). Image scanning was performed with GeneSys software version 1.8.0 (https://www.syngene.com/software/genesys-rapid-gel-image-capture/). Densitometry of all blots was done with the help of ImageJ software (version 1.50, NIH, USA; https://imagej.nih.gov/ij/download.html) normalized with glyceraldehyde 3-phosphate dehydrogenase (GAPDH) as housekeeping protein.

### Quantitative reverse transcriptase polymerase chain reaction (qRT-PCR)

Method published by Khan et al*.*^65^ was used for qRT-PCR to assess the mRNA expression of NLRP3, caspase-1, IL-1β, IL-18 and IL-6 genes. TRI Reagent (Sigma-Aldrich) was used to isolate total RNA. The concentration of the RNA was determined by spectrophotometer (NANO-DROP 1000 VERSION 3.7, Thermo Fisher Scientific). Verso cDNA synthesis kit (Thermo Fisher Scientific) was used to synthesize cDNA from 1 µg of total RNA of each sample as per the manufacturers’ protocol. cDNAs were used as templates for individual PCR reactions using specific primer sets (Table 2). Primers were designed using Primer3 program written by the Whitehead Institute. PCR reactions were carried out on LightCycler 480 (Roche Diagnostic Ltd., Rotkreuz, Switzerland) using LIGHTCYCLER 480 SYBR GREEN PCR KIT (Roche Diagnostics, Risch-Rotkreuz, Switzerland). LightCycler 480 software release 1.5.0 SP4 version 1.5.0.39 (Roche Diagnostics; https://www.bioz.com/result/lightcycler%20480%20software%20version%201%205%200%20sp4/product/Roche) was used to perform quantitative PCR analysis. Expression of each transcript in duplicate was performed and quantified by the relative standard curve method and normalized with housekeeping gene (GAPDH). Obtained transcript value for the control after normalization was arbitrarily assigned a value of unity. The mRNA expression was calculated using the − ΔΔCT method^67^.

**Table 2:** List of forward and reverse primers.

Gene and accession number	Primer sequence	Amplicon length
NLRP3	FP 5′ AGCCAGAGTGGAATGACA 3′	179
NM_145827.4	RP 5′ GCAGGTTCTACTCTATCAAG 3′	
Caspase-1	FP 5′ GGACCCTCAAGTTTTGCCCT 3′	161
NM_009807.2	RP 5′ AACTTGAGCTCCAACCCTCG 3′	
IL-1β	FP 5′ GCCTCGTGCTGTCGGACC 3′	113^65^
NM_008361.4	RP 5′ TGTCGTTGCTTGGTTCTCCTTG 3′	
IL-18	FP 5′ TGTGTTCGAGGATATGACT 3′	170
NM_008360.2	RP 5′ CCTCAAAGGAAATGATCTTG 3′	
IL-6	FP 5′ GCTACCAAACTGGATATAATCAGG 3′	76
NM_031168.2	RP 5′ AGGTAGCTATGGTACTCCAGA 3′	
GAPDH	FP 5′ GGAGAGTGTTTCCTCGTCCC 3′	136
NM_001289726.1	RP 5′ ATGAAGGGGTCGTTGATGGC 3′	

### Enzyme-linked immunosorbent assay

The effect of CP and RA on HMGB-1, TLR-4, MyD88 and IL-1R1 protein levels was studied by using ELISA kits (HMGB-1, catalogue # E0523Mo; TLR-4, catalogue # E1663Mo; IL-1R1, catalogue # E2665Mo from Bioassay Technology Laboratory, Shanghai, China and (MyD88, catalogue # CSB-E12109m from Cusa Biotechnology, Wuhan, China). A complex was formed by mixing the enzyme–antibody conjugate with the mouse kidney homogenate. The strength of the color was directly proportional to the concentration of HMGB-1, TLR-4, MyD88 and IL-1R1 in the kidney homogenate. Optical density was measured at 450 nm by using an ELISA plate reader (BIOTEK POWER WAVE XS2).

### Protein assay

The protein concentration in samples was measured using BSA as standard following the method of Bradford^66^.

### Statistics

The statistical analysis was performed using GRAPHPAD PRISM5 SOFTWARE VERSION 5.04 and VERSION 6.0c (San Diego, CA USA; https://www.graphpad.com/scientific-software/prism/). Results are expressed as means ± standard error of the means (SEM) and one-way analysis of variance (ANOVA) was used with Tukey’s post hoc test to analyze the data. Mann–Whitney *U* test was used to analyze the histopathological scoring^54^. Immunohistochemical data were analyzed by the Kruskal–Wallis test (ANOVA on Ranks) followed by the Dunn's multiple comparison test and values in this case are presented as interquartile range with minimum to maximum.  *P* < 0.05 was considered statistically significant^64^.

### Ethical approval

Study protocols were duly approved by the Institutional Animal Ethical Committee accredited by the Committee for Purpose of Control and Supervision of Experiments on Animals, Government of India which has adopted the ARRIVE Guidelines. All procedures performed in studies involving animals were in accordance with the ethical standards of the institution at which the studies were conducted, as detailed in “Materials and methods”. The manuscript does not contain clinical studies or patient data or any studies with human participants performed by any of the authors.

## Supplementary Information

Supplementary Information.

## Data Availability

No datasets were generated or analyzed during the current study.

## Abbreviations

AKI: Acute kidney injury
CP: Cisplatin
GSH: Reduced glutathione
NF-κB: Nuclear factor-κB
NLRP3: Nucleotide-binding oligomerization domain (NOD)-like receptor containing pyrin domain 3
RA: Rosmarinic acid
IL: Interleukin
