# Supplementary material for: NLRP3 inflammasome in rosmarinic acid-afforded attenuation of acute kidney injury in mice
Source: Sci Rep. 2022 Jan 25;12:1313. doi: 10.1038/s41598-022-04785-z (PMC8789898; doi:10.1038/s41598-022-04785-z)
Supplement: Supplementary file 1 — Supplementary Information. [file 41598_2022_4785_MOESM1_ESM.docx]

**<Scientific Reports>**

**NLRP3 inflammasome in rosmarinic acid-afforded attenuation of acute kidney injury in mice**

Juheb Akhter^1^, Jasim Khan^1$^, Madhu Baghel^3^, Mirza Masroor Ali Beg^1^, Poonam Goswami^1^, Mohd Amir Afjal^1^, Shahzad Ahmad^1^, Haroon Habib^1^, AbulKalam Najmi^2^, Sheikh Raisuddin^1, *^

^1^*Molecular Toxicology Laboratory, Department of Medical Elementology and Toxicology, JamiaHamdard (Hamdard University), New Delhi 110062, India*

^2^*Department of Pharmacology, School of Pharmaceutical Education and Research, JamiaHamdard (Hamdard University), New Delhi, India.*

*^3^Metabolic Research Laboratory, National Institute of Immunology, New Delhi 110067, India*

*^$^Current address: School of Medicine, University of Alabama at Birmingham, Birmingham, Alabama 35233, USA*

Short title: *NLRP3 inflammasome in acute kidney injury*

*Correspondence to: S. Raisuddin, Tel.: +91 11 26059688; Fax: +91 11 26059663.

E-mail address: sraisuddin@jamiahamdard.ac.in (S. Raisuddin)

Supplementary Figure S1

**Biochemical assay results**


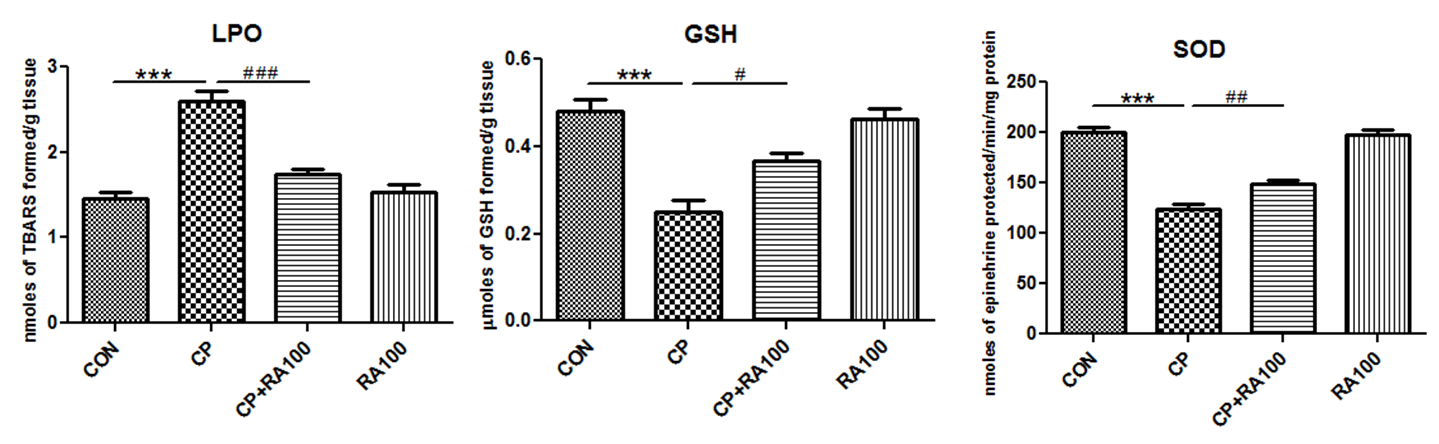


**Legend to Supplementary Figure S1. The effect of RA and CP on the levels of LPO, and GSH and SOD activity.** A single dose of CP caused a significant (****p* < 0.001) increase in the level of LPO and significant (****p* < 0.001) decrease in the level of GSH and SOD when compared with control. RA treatment in CP-treated animals caused a significant (###*p* < 0.001), (#*p* < 0.05) and (##*p* < 0.01) restoration in LPO, GSH and SOD, respectively when compared with the CP-treated animals. RA alone caused no significant change. Data are expressed as means + standard error of means (SEM) (*n* = 6).

**Table S1: Details of the primary and secondary antibodies used in the study**

|  | Catalogue No. | Company | Species | Diluent | Dilution |
| --- | --- | --- | --- | --- | --- |
| Primary antibodies | | | | | |
| NLRP3 | DF7438 | Affinity Biosciences, Cincinnati, OH, USA | H, M, R | 5% skimmed milk in PBST | WB 1:1000 |
| *p*20 Caspase-1 | AF4005 | Affinity Biosciences, Cincinnati, OH, USA | H, M, R | 5% skimmed milk in PBST PBS | WB 1:1000  IHC 1:100 |
| *P*10 Caspase-1 | AF4022 | Affinity Biosciences, Cincinnati, OH, USA | H, M, R | 5% skimmed milk in PBST PBS | WB 1:1000  IHC 1:100 |
| ASC | E-AB-60228 | Elabscience  Houston, Texas, USA | H, M, R | PBST | WB 1:1000 |
| IL-1β | 12242 | Cell Signaling Technology, Beverly, MA, USA | H, M | PBS | IHC 1:100 |
| Cox-2 | E-AB-70031 | Elabscience  Houston, Texas, USA | H, M, R | PBST | WB 1:1000 |
| NFκB-p65 | 3039 | Cell Signaling Technology, Beverly, MA, USA | H, M, R | PBST | WB 1:1000 |
| NLRP3 | E-AB-70161 | Elabscience  Houston, Texas, USA | H, M | PBS | IHC 1:400 |
| KIM-1 | CSB-PA17109A0Rb | CUSABIO TECHNOLY LLC,  Wuhan, China | M | PBS | IHC 1:50 |
| GAPDH | 5174 | Cell Signaling Technology, Beverly, MA, USA | H M R Mk | PBST | WB 1:1000 |
| Secondary antibodies | | | | | |
| Goat anti-rabbit IgG (H+L)(peroxidase/HRP conjugated) | E-AB-1003 | Elabscience  Houston, Texas, USA | Anti-rabbit | PBS | WB 1:10000  IHC 1:500 |
| Goat anti-mouse IgG(H+L)(peroxidase/HRP conjugated) | E-AB-1001 | Elabscience  Houston, Texas, USA | Anti-mouse | PBS | WB 1:10000  IHC 1:500 |

**Abbreviations:** H - human, M - mouse, Mk - monkey, R - rat, WB - western blotting, PBS - phosphate buffer saline, PBST - PBS with Tween 20, IHC - immunohistochemistry

Supplementary Figure S2


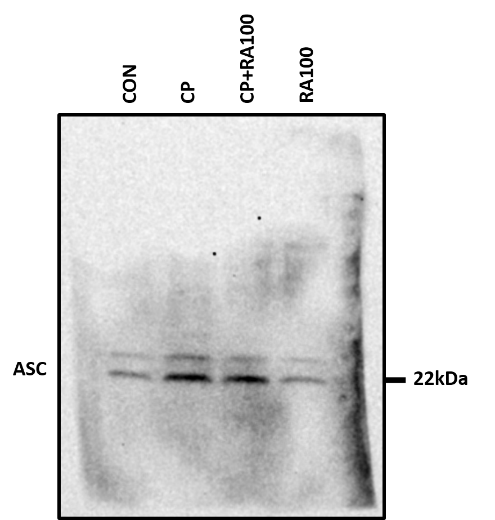
 **
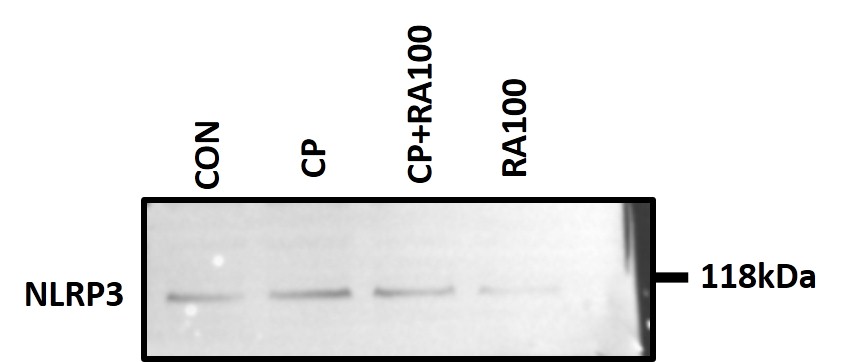
**


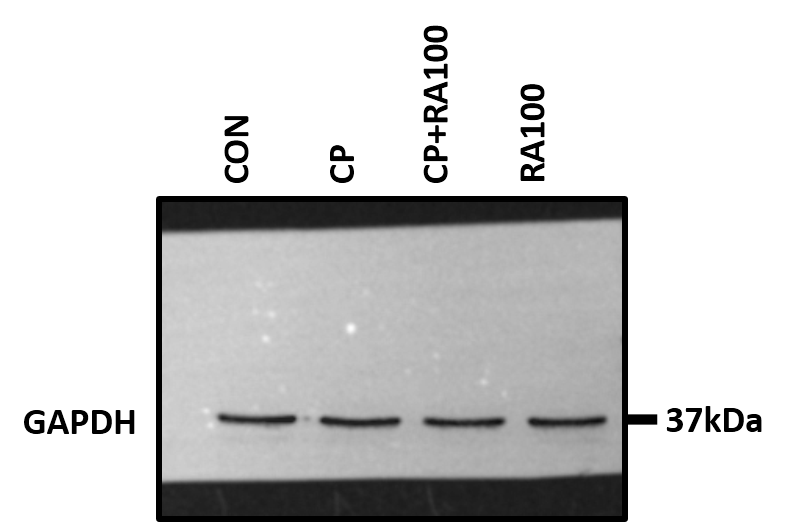


**Legend to Supplementary Figure S2.** Uncropped blot images of corresponding NLRP3, ASC and GAPDHwestern blot images shown in Figure 4.

Supplementary Figure S3


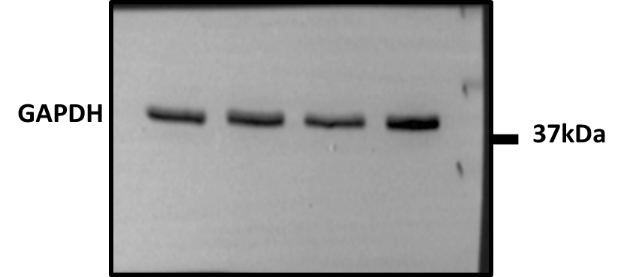

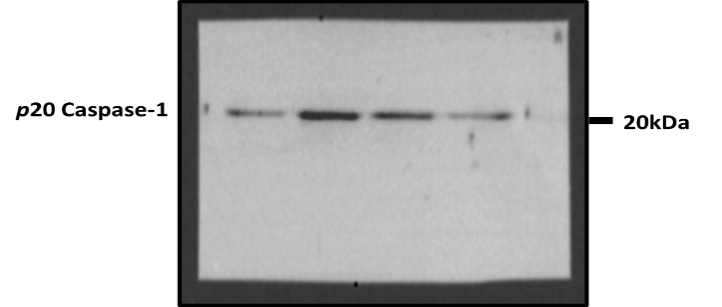

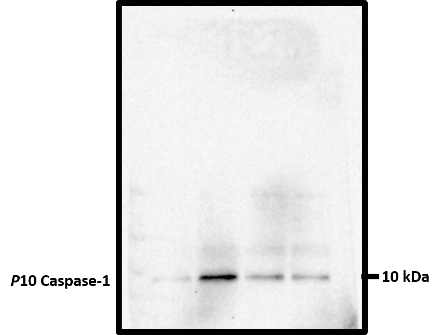


**Legend to Supplementary Figure S3.** Uncropped blot images of corresponding *p*10 Caspase-1, *p*20 Caspase-1 and GAPDHwestern blot images shown in Figure 5.

Supplementary Figure S4


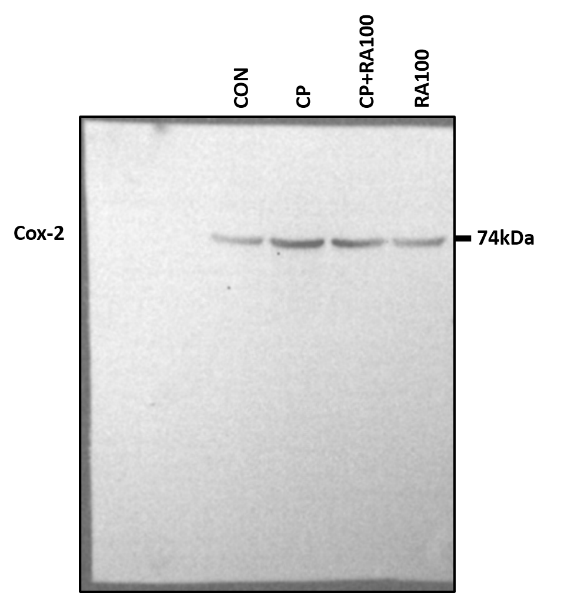

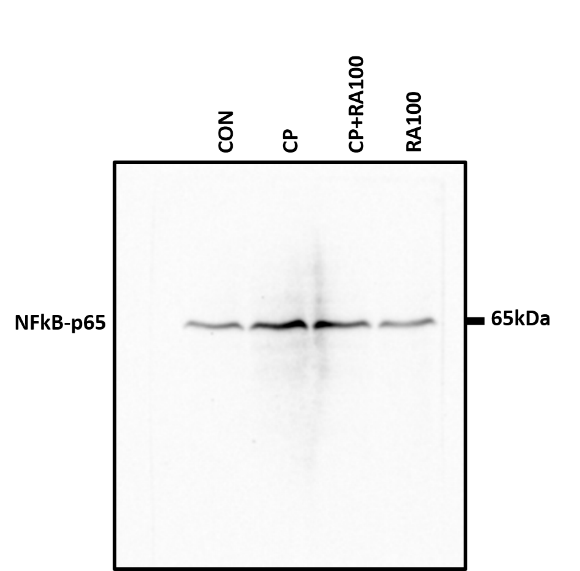


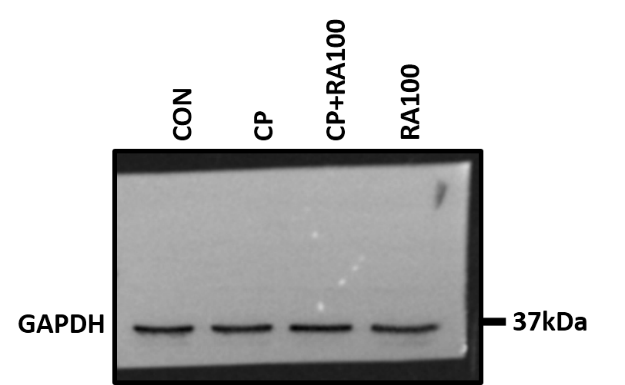


**Legend to Supplementary Figure S4.** Uncropped blot images of corresponding Cox-2, NFƙB-p65 and GAPDH western blot images shown in Figure 6.
